# Beneficial effects of non-matched allogeneic cord blood mononuclear cells upon patients with idiopathic osteoporosis

**DOI:** 10.1186/1479-5876-10-102

**Published:** 2012-05-21

**Authors:** Jun Li, Li Zhang, Liang Zhou, Zheng-Ping Yu, Feng Qi, Bei Liu, Su-Xia Zi, Li Li, Yi Li, San-Bin Wang, Zheng-Jiang Cui, Xing-Hua Pan

**Affiliations:** 1 Stem cell, Tissue and Organ Engineering Research Center, Kunming General, Hospital of Chinese People's Liberation Army, Kunming, 650032, China; 2 Medical school, Kunming University, Kunming, 650214, China; 3 Department of Gynecology and Obstetrics, Kunming General Hospital of Chinese People's Liberation Army, Kunming, 650032, China; 4 Department of Cardiology, Kunming General Hospital of Chinese People's Liberation Army, Kunming, 650032, China; 5 Department of Labour Hygiene, School of Military Preventive Medicine, Third Military Medical University, Chongqing, China; 6 Department of Respiratory Medicine, Kunming General Hospital of Chinese People's Liberation Army, Kunming, 650032, China; 7 Department of Hematology and Endocrinology, Kunming General Hospital of Chinese People's Liberation Army, Kunming, 650032, China; 8 Class 1, Grade 2010, Clinical laboratory technology major, Medical school, Kunming University, Kunming, 650214, China

**Keywords:** Osteoporosis, Cord blood mononuclear cells

## Abstract

**Background:**

Immunological arguments and historical examples have shown that treatment with cord blood for non-hematopoietic activities, such as growth factor production and stimulation of angiogenesis, may not require matching or immune suppression.

**Methods:**

To study the benefit of blood mononuclear cell therapy, 8 patients with idiopathic osteoporosis were given intermittent treatments with non-matched allogeneic cord blood mononuclear cells for 3 months. Morning fasting samples were collected for measuring urine N telopeptide of type-1 collagen, serum bone-specific alkaline phosphatase, and insulin-like growth factor 1 during one-year study.

**Results:**

Clinical response was striking. Serum insulin-like growth factor 1 significantly increased in all patients at 3 months compared with baseline values, from 264.1 ± 107.0 to 384.4 ± 63.1 ng/mL (*P* = 0.002), with a tendency to return to baseline values at 12 months (312.9 ± 75.5 ng/mL, *P* = 0.083). In contrast, differences in serum bone-specific alkaline phosphatase and urine N telopeptide of type-1 collagen were not significant at 3 (*P* = 0.765, *P* = 0.057) or 12 months (*P* = 0.889, *P* = 0.122). A beneficial effect on bone density was observed in all patients at the lumbar spine. The mean bone mineral density calculated during therapy (0.6811 ± 0.1442 g/cm^2^) tended higher than baseline values (0.6239 ± 0.1362 g/cm^2^, *P* < 0), and percentage change (median) varied from 8.85% at 3 months to 7.85% at one year. All patients are now well after one year.

**Conclusions:**

The findings indicate that for these patients with idiopathic osteoporosis, treatment with cord blood mononuclear cells led to a significant increase in insulin-like growth factor 1 levels, which favors the increase in bone mineral density.

## Background

Idiopathic osteoporosis is a rare condition that affects both sexes, women before menopause and men up to sixty-five years. Patients have a decreased ability to recover or regenerate bone mineral content, which may result in bone fracture with little trauma. Diagnosis requires the exclusion of all potential causes of secondary osteoporosis. Asian people on average have a lower bone mineral density than other races, yet their bones fracture less easily; human races also differ in the reference values of bone turnover biomarkers.

Inadequate peak bone mass [1] and increased bone loss are probably involved in the pathogenesis of idiopathic osteoporosis [2]. Both phenomena are associated with low concentrations of insulin-like growth factor 1 in serum [3,4] and bone [5]. The causes of low serum insulin-like growth factor 1 levels in patients with idiopathic osteoporosis remain unclear.

Bone regeneration process involving mesenchymal stem cell invasion, chondrogenesis, osteogenesis, and angiogenesis, breaks down during idiopathic osteoporosis. Circulating multipotent stem cells with characteristic of osteogenic ability have raised interest in novel therapies for osteoporosis [6]. Cord blood mononuclear cells consist of hematopoietic and mesenchymal stem cells, endothelial progenitor cells, and immature immunological cells [7]. Preclinical studies have demonstrated the efficacy of cord blood in the treatment of diverse conditions including amyotrophic lateral sclerosis [8], type I diabetes [9], post-infarct regeneration [10], liver failure [11], and cerebral palsy [12]. Under the practice of medicine, some treatment facilities have been using cord blood stem cells without matching [13-17]. In immune competent recipients, non-matched allogeneic cord blood cells do not elicit graft versus host disease [18-23].

The mesenchymal content of cord blood may explain the tolerogenic capabilities. Cord blood-derived mesenchymal stem cells secrete immune inhibitory cytokines including IL-10 and TGF-β [24,25]. Lymphocytes from cord blood, in contrast to adult blood, are generally immature and usually do not secrete as many inflammatory cytokines [18].

For non-hematopoietic applications, the therapeutic activities of the cord blood are mediated by paracrine effect [26]. In these situations permanent graft survival and matched cells are not essential[17]. The aim of this study is to investigate the benefits of intermittent subcutaneous injections of non-matched allogeneic cord blood mononuclear cells in patients with idiopathic osteoporosis, by evaluating bone density.

## Methods

### Patient characteristics

No prisoner or organs from a prisoner were used in the study. Eight Han Chinese patients (2 men and 6 women, 36–53 years old) with idiopathic osteoporosis were included in this study, selected patients seen at Kunming General Hospital of Chinese People's Liberation Army. The osteoporosis was considered idiopathic after extensive clinical and laboratory investigation that excluded endocrine disorders and other chronic diseases that could cause bone loss. All men and three women had previous fragility fractures at the time of diagnosis, while the others were considered osteoporotic on bone densitometry. All patients were in stable clinical condition at the time of the study, without 1) prior history of severe allergic reactions; 2) history of, or active, malignancy; 3) active systemic or severe focal infections; 4) cardiac, pulmonary, renal, hepatic or gastrointestinal disease; 5) psychiatric disorder; or 6) any immunodeficiency disease or condition (as these could influence the prognosis and end-point measurements).

The patients received conventional treatments without increase in bone mineral density ≥ 1 year prior to entering the current study. They were treated from December 2010 to January 2012 at Kunming General Hospital of Chinese People's Liberation Army. The local institutional review board of Kunming General Hospital of the Chinese People's Liberation Army, under the auspices of the National Ministry of Heath, approved application of the technique. Each patient signed an informed consent form before initiation of treatment. A summary of patients’ clinical history and the results of the first bone density are shown in Table 1.

**Table 1:** Summary of clinical history and the first densitometry data

	P1	P2	P3	P4	P5	P6	P7	P8
sex and age at study (years)	M, 53	M, 41	F, 36	F, 39	F, 41	F, 44	F, 42	F, 37
fragility fractures	vertebral bodies at age 51	vertebral bodies at age 40	vertebral bodies at age 33	right hip at age 36	no	no	since age 33, left humerus	no
age at first densitometry (years)	51	40	33	36	38	41	41	34
LS BMD at first densitometry (g/cm^2^)	0.722	0.617	0.730	0.665	0.589	0.578	0.424	0.608

P: Patient, M: male, F: female, LS: lumbar spine; BMD: bone mineral density

### Cell processing

Umbilical cord blood was collected from healthy unrelated full-term uterine-incision delivery donors (who provided written informed consent) in accordance with the sterile procurement guidelines for cord blood. After collection, each blood sample was tested and verified free of communicable diseases including the hepatitis B and C viruses (HBV and HBC, respectively), human immunodeficiency virus (HIV), alanine aminotransferase (ALT), and syphilis. Testing was performed by a third party laboratory under local government-monitored conditions. Then 2–3 blood samples were mixed together.

Twenty-five milliliters of cord blood was added to 25 mL of Ficoll-Paque PREMIUM 1.073 (GE Healthcare AB, Uppsala, Sweden, http://www.amersham.com) in every 50 mL centrifuge tube and then centrifuged (750 *g,* 25 min). Mononuclear cells were collected and washed twice in saline. Contaminating erythrocytes were lysed with lysis buffer consisting of injection grade water. The cells were sieved through a 100 eye cell sieve mesh before administration and were injected as quickly as possibly after preparation (cell number ≥6 × 10^7^, containing 0.5% CD34^+^ cells, 0.2% CD11b^+^ cells, 9.7% CD4^+^ cells, and 8.6% CD14^+^ cell as determined by flow cytometry; cell viability ≥96%).

### Cell administration

Each patient was treated intermittently with 6 × 10^7^ cells/dose, at 4-weeks intervals, 4 times in total, with a follow-up period of 12 months. Twenty milliliters of cell suspension in saline solution (6 × 10^7^ cells/20 mL) were subcutaneously injected in several places in the left arm of every patient for convenient observation over 10 to 20 minutes. Injection was repeated 4 times, treatments were separated at 4 week-intervals, and each time with different baby-derived cord blood cells (each time with 2–3 cord blood cell samples for every patient). All patients received 10 samples of cord blood cells in total. After each dose, patients were asked if they experienced discomfort or improvement of syndrome before administering the next dose. No patient received any other medicine, to avoid an interference effect during the therapy schedule (the entire year).

### Routine test, insulin-like growth factor 1, bone-specific alkaline phosphatase, N telopeptide of type-1 collagen and bone mineral density measurements

Patients returned monthly for clinical examination that included blood pressure and body weight measurements. Blood was drawn after overnight fasting for routine tests. Serum calcium, phosphate, parameters of liver function, lipids, and blood glucose were measured via automated techniques. Fasting morning serum samples were collected for serum insulin-like growth factor 1 and bone-specific alkaline phosphatase, and a sample of the second voided urine was collected for evaluation of the N-telopeptide of type-1collagen at baseline, 3 months and 12 months after the initial treatment. Concentration of insulin-like growth factor 1 was measured with the kit DSL 5600 ACTIVE, EUA. Bone-specific alkaline phosphatase was measured with the kit Alkphaseb, USA. Urinary N-telopeptide of type-1collagen was measured with the kit Osteomark, USA.

Bone mineral density was measured at the lumbar spine using dual-energy X-ray absorptiometry (Expert, Lunar, Madison, WI, USA) at baseline, 3 months and 1 year after the initial treatment. Percent variations in bone mineral density ≥1.5% at the lumbar spine were considered significant. Statistical analysis was performed using the software IBM SPSS 18.0. One-way ANOVA for repeated measures and the paired *t*-test were used to evaluate the differences between levels at baseline and at 3 months, and between baseline and at 1 year. *P-*values < 0.05 were considered significant.

## Results

Analysis of overall effect for a one year follow-up (beginning with the first administration) showed that treatment with cord blood mononuclear cells via subcutaneous injections was well-tolerated. No allergic or immunological reactions, graft-versus-host disease, or serious adverse effects were observed at the time of injection or while under observation. All patients were free of communicable diseases such as HBV, HCV, HIV, ALT, and syphilis at 3 months. None of the patient had to be withdrawn from the study. Blood pressure and body mass index did not change, nor did the parameters of renal and hepatic functions, serum calcium, phosphate and lipids.

Serum insulin-like growth factor 1 increased in all patients and remained above baseline values during treatment. The mean insulin-like growth factor 1 calculated during the therapy (348.6 ± 66.9 ng/mL) tended to be higher than baseline values (264.1 ± 107.0 ng/mL, *P* = 0.008). The mean serum insulin-like growth factor 1 increased at 3 months from 264.1 ± 107.0 to 384.4 ± 63.1 ng/mL (*P* = 0.002) and tended to return to baseline values at 1 year (312.9 ± 75.5 ng/mL, *P* = 0.083). The difference in serum insulin-like growth factor 1 at 3 months and 1 year of the therapy schedule was also significant (*P* = 0.001). Individual values are shown in Table 2.

**Table 2:** Individual values of insulin-like growth factor 1 (ng/mL) along the therapy

	Baseline	3 months	1 year
IGF-1 (ng/ml) *			
Patient 1 (male)	286	372	390
Patient 2 (male)	277	388	286
Patient 3 (male)	183	306	232
Patient 4 (male)	365	446	359
Patient 5 (male)	406	489	428
Patient 6 (male)	89	371	280
Patient 7 (male)	177	304	212
Patient 8 (male)	330	399	316

* *p* = 0.002 comparing 3 months to baseline, *p* = 0.083 comparing 1 year to baseline

The mean serum bone-specific alkaline phosphatase levels did not change remarkably: it increased from 16.2 ± 8.0 U/L at baseline to 16.5 ± 6.3 U/L at 3 months, and to 15.9 ± 4.2 U/L at 12 months, but the variations were not significant (*P* = 0.765, *P* = 0.889, respectively). The mean urine N telopeptide of type-1 collagen decreased from 16.8 ± 4.3 nMBCE/mMCr at baseline to 13.8 ± 2.4 nMBCE/mMCr at 3 months, and tended to return to baseline values at 12 months (14.8 ± 4.0 nMBCE/mMCr), but variations were not significant (*P =* 0.057, *P =* 0.122, respectively).

**Figure 1:**
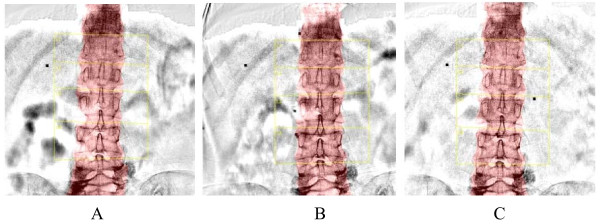
**The lumbar spine (L2-L4) bone mineral density of patient 7 at baseline**. **A**, The lumbar spine (L2-L4) bone mineral density of patient 7 at baseline. **B**, The lumbar spine (L2-L4) bone mineral density of patient 7 at 3 months. **C**, The lumbar spine (L2-L4) bone mineral density of patient 7 at 1 year.

Absolute values of bone mineral density at baseline, 3 months and 1 year are shown in Table 3. A beneficial effect on bone density was observed in all of the patients at the lumbar spine with therapeutic schedules of treatment after either 3 or 12 months, and percentage change (median) varied from 8.85% at 3 months to 7.85% at one year. To estimate the effect of cord blood mononuclear cells, the bone density after therapy was compared with that at baseline (Figure 1). The mean bone mineral density calculated during the therapy (0.6811 ± 0.1442 g/cm^2^) tended to be higher than baseline values (0.6239 ± 0.1362 g/cm^2^, *P* < 0). The mean bone mineral density varied from 0.6239 ± 0.1362 at baseline to 0.6836 ± 0.1444 (*P* < 0) at 3 months and tended to return to baseline values at 1 year (0.6785 ± 0.1441, *P* < 0); the variation in bone density from 3 months to 1 year of the therapy schedule was also significant (*P* = 0.005). The effect of cord blood mononuclear cells was positive in all patients at the lumbar spine, as showed a net variation of > 1.5%.

**Table 3:** **Variation and percent change in lumbar spine bone mineral density (g/cm**^**2**^**)**

	P 1	P 2	P 3	P 4	P 5	P 6	P 7	P 8
	(M)	(M)	(F)	(F)	(F)	(F)	(F)	(F)
LS BMD (g/cm^2^) at baseline	0.707	0.611	0.790	0.623	0.784	0.455	0.423	0.598
LS BMD (g/cm^2^) at 3 months	0.761	0.668	0.884	0.650	0.846	0.493	0.495	0.672
LS BMD (g/cm^2^) at 1 year	0.749	0.660	0.879	0.650	0.844	0.488	0.491	0.667
% change LS BMD at 3 months	7.6	9.3	11.9	4.3	7.9	8.4	17.0	12.4
% change LS BMD at 1 year	5.9	8.0	11.3	4.3	7.7	7.3	16.1	11.5
Effect on LS BMD	+	+	+	+	+	+	+	+

P: Patient, (M): male, (F): female, LS: lumbar spine, BMD: bone mineral density, +: positive. The effect of cord blood mononuclear cells was considered as positive when the increase in bone mineral density was bigger than 1.5% at lumbar spine or when patients stopped loosing bone

## Discussion

Although cord blood-derived cells are superior to bone marrow in terms of growth factor production ability, pluripotency, and immune modulating activity [27], their use has been limited to autologous sources for regenerative applications [17]. In the present study we administrated the latest extracted cells without any other medicine to avoid their interference effect during the whole year. No graft versus host disease or adverse effects were observed after treatment. All patients were free of communicable diseases such as HBV, HCV, HIV, ALT, and syphilis at 3 months. We believe that cord blood mononuclear cells are a viable alternative source of stem cells as they are safely applicable without significant adverse effects.

All patients had a net bone gain of > 1.5% at the lumbar spine with the therapy in the current study. The variations in serum bone-specific alkaline phosphatase and urine N telopeptide of type-1 collagen were not significant at 3 or 12 months. However, the mean urine N telopeptide of type-1 collagen decreased at 3 months and tended to return to baseline values at 12 months (*P* = 0.057, *P* = 0.122). The variations in bone turnover marker during therapy usually take place far earlier than those of the bone density. The time points of 3 months and 12 months and the small sample size in this study may contribute to the negative result for N telopeptide of type-1 collagen; and the serum bone-specific alkaline phosphatase levels did not change remarkably, indicating that the possible effect of cord blood mononuclear cells is primarily antiresorptive.

We maintained cord blood mononuclear cells during the first 3 months of therapy. The monthly dose of 6 × 10^7^ cord blood mononuclear cells increased insulin-like growth factor 1 levels in all patients at 3 months, and eventual supernormal levels were sustained after 1 year, although the difference at 12 months was not significant. The maintenance of insulin-like growth factor 1 concentrations in the blood correlated with bone density increase in response to cord blood mononuclear cell therapy. Insulin-like growth factor 1 influenced the effect of cord blood mononuclear cells on bone. The data suggest that cord blood mononuclear cells contributed to the increase in insulin-like growth factor 1 concentrations in blood during the treatment, thus favoring bone mineral density increase. Insulin-like growth factor 1 response to cord blood mononuclear cells probably contributed to the normalization of bone formation. It initializes bone remodeling cycles with a relatively greater enhancement of bone density, probably explaining the further increase in bone density observed in this study after stopping cord blood mononuclear cells in patients with idiopathic osteoporosis. As a consequence, the expected beneficial effect of cord blood mononuclear cells on bone density already was evident after the first year.

The difference in bone density between 3 months and 1 year of the therapy schedule was significant (*P =* 0.005). An attenuated positive effect on lumbar spine bone density was seen after 1 year, as insulin-like growth factor 1 was maintained above baseline values, although the difference in insulin-like growth factor 1 between baseline and 1 year was not significant. Thus, it is possible that our patients will continue to decrease bone density in the following year.

Although cord blood stem cells can differentiate into diverse cell lineages [28], the injected cells stimulate the intrinsic stem cells to help them proliferate and differentiate [29]. Not the replacement of injured tissue by transdifferentiation of stem cells, but the paracrine effect promotes the regeneration of damaged tissue [30,31]. We assume that the paracrine effect mainly contributed to our patient’s improvements in this study.

Clinical effect of cord blood transplantation depends on the mononuclear cells number, but most cord blood samples do not contain enough stem cells. Increasing cell dose improves engraftment, and partially surmount the influence of human leukocyte antigen disparities. Since 2005, the number of adult patients transplanted with double umbilical cord blood has surpassed the number of adults receiving single cord blood units [32]. In this study we injected the cord blood mononuclear cells expecting transient engraftment and stimulation (paracrine) activity. We administrated the cells from a mixture of several blood samples to guarantee the engraftment of stem cells [33,34]. At least 6 × 10^7^ cells were given each time, enough for cell transplantation, and at the same time we administrated the intermittent treatment with 6 × 10^7^ cells/dose for safe opinion. The repeated administration (4 times) maintained the survival of enough of the stem cells in the patients’ bodies. The prolonged infusion intervals (4 weeks) can prevent immune responses induced by repeated administration.

Some studies demonstrated that stem cell therapy can result in functional improvement of the damaged organs in long term period [35,36]. Although we cannot guarantee the longer term effect from our study alone, the results from this long term study are encouraging. One limitation of this study is the small sample size, but idiopathic osteoporosis is an uncommon disease. The other limitation is the absence of a placebo-treated control group. However, the patients received conventional treatments without significant increase in bone mineral density prior to entering the current study. In addition, the effect of cord blood mononuclear cells can be considered positive when the increase in bone mineral density is greater than 1.5% at the lumbar spine, or when patients stop losing bone. For this purpose, each patient could be considered as his own control.

## Conclusion

Our data suggest that intermittent administration of non-matched allogeneic cord blood mononuclear cells therapy exerts beneficial effects on bone mineral density in patients with idiopathic osteoporosis. Insulin-like growth factor 1 initializes bone remodeling cycles with a greater enhancement of bone density, probably explaining the increase in bone density observed in this study.

The importance of this study is that it is the first to investigate the effects of non-matched allogeneic cord blood mononuclear cells on bone mineral density in patients with idiopathic osteoporosis, and the positive effect is encouraging. More studies using this regimen for longer periods are needed to further define the effects of cord blood mononuclear cells on bone density and fracture risk in patients with idiopathic osteoporosis.

## Competing interests

The authors declare that they have no competing interests.

## Authors' contributions

JL conceived the study, participated in its design and coordination, carried out the clinical treatment and performed the statistical analysis. Li-Z analyzed and interpreted data and drafted the manuscript. Liang-Z, ZY, FQ, BL, SZ, LL, YL carried out the clinical treatment and collected data. SW, ZC analyzed and interpreted data and helped to draft the manuscript. XP conceived the study, participated in its design and coordination and helped to draft the manuscript. All authors read and approved the final manuscript.
